# Single Particle Combustion of Pre-Stressed Aluminum

**DOI:** 10.3390/ma12111737

**Published:** 2019-05-29

**Authors:** Kevin J. Hill, Michelle L. Pantoya, Ephraim Washburn, Joseph Kalman

**Affiliations:** 1 Department of Mechanical Engineering, Texas Tech University, Lubbock, TX 79409, USA; kevin.hill@ttu.edu (K.J.H.); michelle.pantoya@ttu.edu (M.L.P.); 2 Combustion Science and Propulsion Research Branch, Naval Air Warfare Center Weapons Division, 1 Administration Circle, China Lake, CA 93555, USA; ephraim.washburn@navy.mil; 3 Department of Mechanical and Aerospace Engineering, California State University, Long Beach, Long Beach, CA 90840, USA

**Keywords:** aluminum, stress, strain, laser ignition, reaction mechanism, solid fuels

## Abstract

An approach for optimizing fuel particle reactivity involves the metallurgical process of pre-stressing. This study examined the effects of pre-stressing on aluminum (Al) particle ignition delay and burn times upon thermal ignition by laser heating. Pre-stressing was by annealing Al powder at 573 K and quenching ranged from slow (i.e., 200 K/min) identified as pre-stressed (PS) Al to fast (i.e., 900 K/min) identified as super quenched (SQ) Al. Synchrotron X-ray Diffraction (XRD) analysis quantified an order of magnitude which increased dilatational strain that resulted from PS Al and SQ Al compared to untreated (UN) Al powder. The results show PS Al particles exhibit reduced ignition delay times resulting from elevated strain that relaxes upon laser heating. SQ Al particles exhibit faster burn times resulting from delamination at the particle core-shell interface that reduces dilatational strain and promotes accelerated diffusion reactions. These results link the mechanical property of strain to reaction mechanisms associated with shell mechanics that explain ignition and burning behavior, and show pre-stressing has the potential to improve particle reactivity.

## 1. Introduction

Aluminum (Al) is an important solid fuel for propulsion and pyrotechnic applications because of its high (85 GJ/m^3^) stored chemical energy, and its burning behavior at a range of particle sizes and environments are of great interest. For propulsion applications, ignition delay and burn times of Al particles are important to understand because reduced Al particle burn time and ignition delay can provide added energy to facilitate propellant surface burning regression or reduce particle agglomeration on the burning propellant surface [1]. A promising approach to improve Al particle ignition delay and burn time is pre-stressing [2,3]. Pre-stressing is the intentional creation of favorable stresses in core-shell Al particles via annealing and quenching. The induced stresses place the shell in compression, and change the dynamics of shell failure during ignition and subsequent combustion. Considerable studies have been undertaken on pre-stressed Al particles under impact [2,3], but no studies have been conducted on the combustion of pre-stressed Al particles under thermal loads for single particle conditions.

Single particle combustion of Al has a long history of experimental and theoretical work [4,5,6,7,8,9]. Several systems exist for creating well-dispersed dust clouds of single particles for combustion studies. Methods include fluidized bed elution [10,11], vortex mixing [12], electrostatic aerosolization [8,9], and others [13,14]. The goal of each system is to create clouds of particles with stable concentrations and particle sizes. These systems have not been used to study pre-stressed Al particles. In this study, 800 µm diameter glass beads were used as a fluidizing medium to create a low-concentration stream of 3–4.5 µm diameter Al particles (particle size has previously been reported in [15]). The eluted particles were then heated and ignited with a 600 W CO_2_ laser. The objective was to examine the influence of pre-stressing on ignition delay and burn times under thermal initiation conditions.

## 2. Materials and Methods

Aluminum particles (3–4.5 µm diameter, Alfa Aesar, Haverhill, MA, USA) were treated through an annealing and quenching process that is detailed elsewhere [2,3] but summarized here. Pre-stressing aluminum (PS Al) particles was undertaken by heating at 10 K/min to 573 K, holding isothermally for 15 min, and quenching at a rate of 200 K/min via a TA Instruments Q800 DMA (dynamic mechanical analyzer, TA Instruments, New Castle, DE, USA). For faster quenching, super quenched aluminum (SQ Al) powders were placed in a custom water-tight quenching chamber, held isothermally at 573 K for 15 min in a Ney bake-out oven, and quenched at a rate of 900 K/min via a brine bath composed of water, salt, surfactant, and dish soap [3]. It is notable that the powder was never in direct contact with the liquid solution used for quenching.

### 2.1. Synchrotron X-Ray Diffraction (XRD) Measurements

The PS Al, SQ Al, and untreated Al (UN Al) powders were analyzed using Synchrotron XRD at the Advanced Light Source facility at Lawrence Berkeley National Laboratory on beamline 12.3.2. This beamline uses a micron focused synchrotron X-ray beam to determine dilatational strain and a white micro-beam technique [16,17] to determine dilatational strain data that was previously reported [3,15,18,19].

### 2.2. Single Particle Reactive Characterization

**Figure 1:**
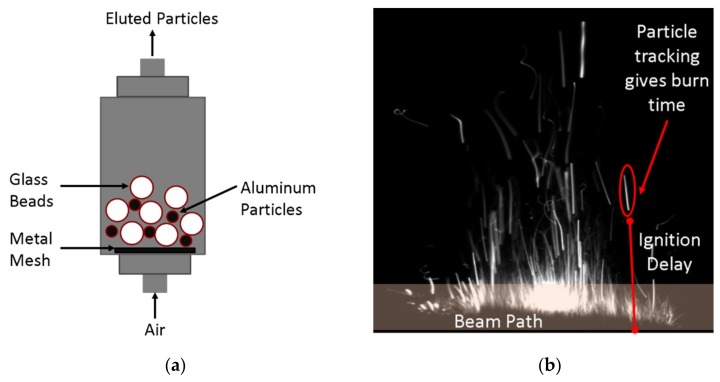
(**a**) Schematic of particle injection system showing Al particles and glass beads used to elute Al particles into the laser beam path. (**b**) Photograph of untreated aluminum powder (UN Al) particle burning upon exposure to laser beam representing burn time and ignition delay measurements.

A 1-g powder sample of PS Al, SQ Al, or UN Al powder was loaded into the particle injector shown Figure 1a and composed of a 5.08 cm by 30.48 cm vacuum flange filled with 800 µm of glass beads at a weight ratio of 100:1 beads to Al powder. Dry shop air was passed through the powder bed at a rate of 14.2 L/min, and the eluted particles were passed through the beam path of a 600 W CO_2_ laser focused down to a 200 µm spot size as shown in Figure 1b. The laser was programed to operate in 1 ms pulses with 10 ms between pulses, for a 10% duty cycle. A Photron SA-Z with a K2 microscopic lens (Infinity, Centennial, CO, USA) recording at 30,000 frames/s with a resolution of 1024 × 1024 pixels (64.2 pixels/mm) was triggered with each laser pulse to record particle ignition and combustion. The mean particle velocity was approximately 5.3 m/s (based on volumetric flow rate) with an observation time of 2 ms. An in-house MATLAB^®^ script analyzed burn time and ignition delay data by tracking particle residence time and location of first light respectively, as shown in Figure 1b.

## 3. Results

**Figure 2:**
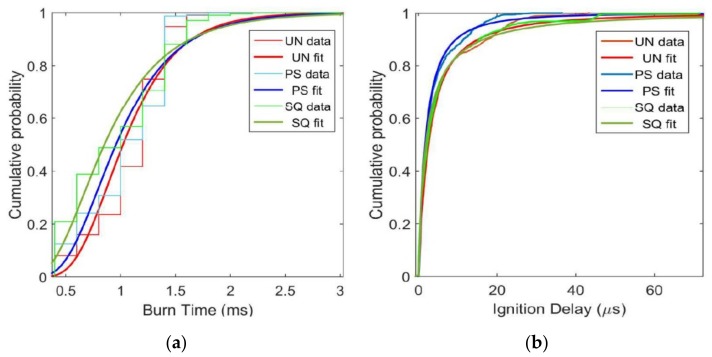
Cumulative probability of: (**a**) burn time and (**b**) ignition delay for untreated Al powder (UN Al), pre-stressed Al powder (PS Al) and super-quenched Al powder (SQ Al).

Figure 2 shows cumulative probability plots for burn time and ignition delay of each material tested and provides statistical insight for large (>5000 particles) sample sizes. The apparent discretization on the burn time plot (Figure 2a) is a result of low time resolution due to a relatively slow frame rate, while the smoother ignition delay plot (Figure 2b) is a result of large spatial resolution. The scatter in the data is a result of the polydisperse particles endemic to the Al powder used.

Table 1 shows the true mean, average for all data within two standard deviations (2 SD), average for all data within three standard deviations (3 SD), and the median for all data, as well as percent differences from UN Al for the PS Al and SQ Al particles. The SQ Al has the fastest burn time, followed by PS Al. The largest percent decrease in burn time compared to UN Al for both PS Al and SQ Al occur when only two standard deviations of data are considered, indicating outliers in the data that may be a function of agglomeration. The PS Al has the largest decrease in ignition delay, with little difference depending on how many standard deviations were considered (i.e., there are few outliers in this data).

## 4. Discussion

Figure 2 and Table 1 show modest improvement in burn time for PS Al and SQ Al compared to UN Al particles. The SQ Al particles show little difference in ignition delay time compared to the UN Al, indicating that combustion begins after a similar amount of laser energy was absorbed. However, PS Al particles show a substantially shorter ignition delay time (60% of the particles ignited 30.6% faster and 90% ignited 36.3% faster than UN Al) (Figure 2). A shorter ignition delay time could occur for larger particle sizes in the population (sizes that require more energy to ignite) if some energy release occurs due to internal stress release during heating [2,3]. However, calculating the strain energy for a single 3.5 µm PS Al particle yields 7 × 10^−15^ J, while the energy required to heat the same particle to the melting temperature of alumina is approximately 1 × 10^−7^ J.

Synchrotron XRD measurements in Table 2 for UN Al, PS Al and SQ Al particles show an increase in dilatational strain within the core-shell particle that results from pre-stressing. The UN Al particles show nearly negligible residual strain, indicating that the process of pre-stressing elevates the strain of the Al particle by an order of magnitude. However, SQ Al particles produce a measurably lower dilatational strain compared with PS Al despite the theoretical prediction that faster quenching will elevate the strain [3]. The reason for the decreased dilatational strain under super quenching conditions has been modeled as delamination at the core-shell particle interface that relaxes induced stresses and reduces dilatational strain within the particle. Delamination at the core-shell interface is predicted to be on the order of 52% for 3–4.5 μm in diameter of SQ Al particles [3]. Figure 2 shows PS Al particles with elevated dilatation strain (Table 2) have shorter ignition delay time than the other samples. In contrast, SQ Al and UN Al have lower strain (Table 2) and therefore, less added energy to reduce ignition delay time. 

The SQ Al particles had the shortest burn times (11.8% faster than UN Al at 60% of total particle population), with burn times for all particle treatments converging at 90% of the total particle population (Figure 2). Shorter burn times indicate a reduced diffusion barrier in the SQ Al particles that may be attributed to shell delamination [3], which could lead to cracking in the alumina shell. Shell cracking would reduce the barrier for diffusion reactions as evidenced by the shorter burn times.

Aluminum particles inherently include a surface hydration layer surrounding the Al_2_O_3_ passivation shell. Annealing and quenching the Al particles may alter the surface hydration layer and affect surface energy forces that promote agglomeration. It is assumed that after annealed and quenched particles were exposed to ambient conditions, they would naturally rehydrate. Visual inspection of the Al powders reveals no apparent differences in agglomeration behavior that would contribute to the behaviors observed in Figure 2.

The results presented in Figure 2 are consistent with previous work on pre-stressed particles under impact loads [3]. Hill et al. [3] reported that PS Al particles demonstrate higher pressurization rates than SQ Al particles under impact loads, and is similar to the reduced ignition delay times of PS Al particles shown in Figure 2 compared with both SQ Al and UN Al particles. Additionally, the reduced particle burn times of SQ Al particles are consistent with the higher peak pressures and increased combustion completeness of SQ Al particles shown in Hill et al. [3].

## 5. Conclusions

Aluminum particles have been pre-stressed using two different quenching treatments that increase their dilatational strain. Quenching slowly elevates the particle strain and quenching quickly promotes delamination at the core-shell particle interface and reduces particle strain. Both quenching treatments produce Al particles at elevated strain compared with untreated Al. The mechanical alteration of the Al particles affects their ignition and burning behavior when subjected to a CO_2_ laser beam. Pre-stressed Al particles quenched slowly (PS Al) exhibit reduced ignition delay times while pre-stressed particles quenched quickly (SQ Al) exhibit reduced burn times. Elevated strain energy may contribute to a reduction of energy needed to ignite the particles thereby leading to reduced ignition delay time. Shell-core delamination associated with SQ Al particles facilitates diffusion reactions thereby reducing burn times. Altering the mechanical properties of the shell-core particles alters their reaction mechanism. This study links reactive behavior with mechanistic understanding associated with the mechanical property of dilatational strain. 

## Figures and Tables

**Table 1:** Average and median burn time and ignition delay values within different standard deviation (SD) bounds (with % differences from the UN particles).

Particle Type	Mean (All Data)	Average (2 SD)	Average (3 SD)	Median
	Burn Time (ms)			
UN	1.08	1.37	1.08	1.2
PS	1.04	1.03	1.03	1
SQ	0.96	0.95	0.96	1
**Particle Type**	**Ignition Delay Time (µs)**			
UN	13.79	9.88	12.42	6.42
PS	9.37	6.80	8.29	4.26
SQ	14.18	10.16	10.80	5.59
**Particle Type**	**Burn Time % Decrease from UN Al**			
PS	4.37	24.86	4.48	16.67
SQ	11.34	30.67	11.47	16.67
**Particle Type**	**Ignition Delay Time % Decrease from UN Al**			
PS	32.11	31.15	33.30	33.67
SQ	−2.82	−2.81	13.07	12.87

**Table 2:** Synchrotron XRD measurements of dilatational strain for 3–4.5 µAl particles [3].

Material	Dilatational Strain
UN Al	1.5 × 10^−6^
PS Al	9.23 × 10^−5^
SQ Al	5.7 × 10^−5^
